# Corrigendum: Carbapenemase-producing *Enterobacteriaceae* (CPE) isolated from pigs in China

**DOI:** 10.1099/acmi.0.000072

**Published:** 2019-12-20

**Authors:** Man-Ki Tong, Melissa Chun-Jiao Liu, Eileen Ling-Yi Lai, Kin-Hung Chow, Pak-Leung Ho

**Affiliations:** ^1^​ The University of Hong Kong, Hong Kong, Hong Kong

This abstract contained reference to the drug ‘azithromycin’ where ‘aztreonam’ should have been stated.

The authors apologise for any inconvenience.

